# Differences in Physical Activity between Patients with Peripheral Artery Disease and Healthy Subjects

**DOI:** 10.1155/2020/5093528

**Published:** 2020-10-20

**Authors:** Sumiko Shiba, Akiko Shiba, Atsutoshi Hatada

**Affiliations:** ^1^Department of Physical Therapy, Konan Women's University, Kobe City 658-0001, Japan; ^2^Department of Nursing, Akisaki Clinic, Wakayama City 640-0002, Japan; ^3^Department of Cardiovascular Surgery, Saiseikai Wakayama Hospital, Wakayama City 640-8518, Japan

## Abstract

**Objectives:**

Peripheral artery disease (PAD) is a significant prognostic marker of poor long-term survival due to limited physical activity associated with various functional problems, such as intermittent claudication. A physically active lifestyle has the potential to modify peripheral artery risk factors and promote general health. While low daily physical activity levels have been recognized in the population of PAD, the exact level has yet to be quantified due to lack of research. The aim of the present study was to compare physical activity level (PAL) and time spent at activities of different intensity levels between patients with PAD and healthy individuals. The study subjects were 10 patients with PAD and 10 age-matched healthy control subjects. We measured the time spent at light, moderate, or vigorous physical activity using triaxial accelerometer and calculated PAL. Intermittent claudication onset distance and maximum walking distance were defined as the distance walked at which the subject first reported leg pain and the distance at which the subject was unable to continue walking because of leg pain, respectively.

**Results:**

Our results showed (i) lower PAL in patients with PAD compared with the controls; (ii) while there was no significant difference in the high-intensity activity between the two groups, the light- and moderate-intensity activities of the PAD group were significantly lower than the controls, the time spent at moderate-intensity activity was approximately 50% less; and (iii) among patients with PAD, low PAL did not correlate directly with intermittent claudication.

**Conclusions:**

PAD patients limit the amount of their physical activity, especially light and moderate intensities. Our study highlights the importance of spending more time walking in daily life.

## 1. Introduction

Peripheral artery disease (PAD) is an atherosclerotic disease known to lead to narrowing of the lower limb arteries. Previous studies reported that PAD is associated with significant risk of cardiovascular disease morbidity and mortality [1–3]. As the primary symptom of PAD, intermittent claudication (IC) appears during physical activity when the blood and oxygen demands of the working skeletal muscle exceed supply. Patients with IC experience functional decline due to the limitation in physical activity, further raising the risk of cardiovascular events in a vascular system already compromised by the underlying atherosclerosis. On the contrary, physical activity and cardiorespiratory fitness are known to be associated with enhanced health and quality of life, and even small improvement in fitness correlates with reduced cardiovascular and all-cause mortality [4–8]. Therefore, it is important to encourage physical activity to improve health status. While low daily physical activity levels have been recognized in this population, the exact level has yet to be quantified due to lack of research. Therefore, it is not clear how much physical activity patients with PAD lack compared to healthy people. In other words, the minimum daily physical activity required by patients with PAD remains undetermined. We hypothesized that patients with PAD spend almost all of their time in a low-intensity state of physical activity and thus are generally lacking in high-intensity physical activity levels compared to healthy people.

Since the methods for rehabilitation treatment for PAD in Japan are less advanced compared to other developed countries, these patients are not provided the appropriate treatment. We hope the present study will lead to insights into effective methods for improving daily physical activity levels. The aim of the present study was to compare the mean physical activity level and the time spent in activities of different intensity levels between PAD patients and healthy controls.

## 2. Materials and Methods

### 2.1. Subjects

Ten patients with PAD and ten healthy individuals of similar age (control group) participated in this study. All patients with PAD were evaluated clinically by a cardiovascular surgeon and underwent the ankle brachial index (ABI) test. We defined PAD as an ABI <0.90. Patients with clinically severe cardiac, pulmonary, musculoskeletal disease that could alter gait, or severe risk of malnutrition were excluded. The control group consisted of subjects who reported no history of claudication and had no evidence of cardiovascular or pulmonary disease or lower extremity musculoskeletal disease known to affect gait. None of the participants performed the rehabilitation therapy for PAD.

The Human Ethics Committee approved the present study and a written informed consent was obtained from all subjects.

### 2.2. Walking Test

All patients with PAD performed the walking test, which consisted of walking a route of 20 meters with turns, delimited by two cones. The subjects were instructed to walk as 12 laps as they could at their comfortable speed and to inform the evaluator of any sudden onset of pain. Intermittent claudication onset distance (ICD) and maximum walking distance (MWD) were defined as the distance walked when the subject first reported leg pain and the distance at which the subject was unable to continue walking due to leg pain, respectively [9, 10]. We asked the subjects to walk at a comfortable speed in order to determine the relationship in their daily walking.

### 2.3. Accelerometer-Measured Physical Activity

Physical activity over a 7-day interval was recorded using a triaxial accelerometer (model: Active Style Pro HJA-750C, Omron Healthcare, Japan). This triaxial accelerometer, in combination with a gravity-removal physical activity classification algorithm, provides estimates of daily physical activity and classification of household and locomotive activities. Data were summed and stored in 10 s epoch length within the range of acceleration data of ±6G and with a resolution of 3 mG. The validity and reliability of the triaxial activity monitor in accurately capturing physical activity have been reported previously [11–13]. Metabolic equivalents (METs) determined by the Active Style Pro correlated significantly with METs measured by indirect calorimetry [11, 12] and provide accurate estimate of energy expenditure under free-living conditions [13]. This monitor provides information on METs, physical activity energy expenditure (PAEE), total energy expenditure (TEE), the number of steps taken, and the amount of time for physical activity.

Subjects were instructed to wear the accelerometer on their waist, continuously throughout the day, removing it only for bathing or swimming and to wear them over eight days. To be included in the study, subjects had to have at least seven valid days, including ≥5 valid weekdays and ≥2 valid weekends. When the subjects forget to reattach the accelerometer, they were asked to wear it for an extended period. If that period included a weekday, they were asked to make up for the lack of data on that day. When the participant forgot to reattach the accelerometer on a weekend, and if the scheduled last next day was a weekday, she was asked to wear it continuously until the end of the next weekend in order to obtain valid data. We emphasized to the participants that they must not change anything in their lifestyle during the experiment's duration. The screen on the triaxial accelerometer was set to blank so that the participants could not check any indicators such as step count or the activity intensity for themselves.

We calculate the average daily data from a seven-day period, in which the following indices were examined (in minutes per day): 1 to <3 METs for low-intensity activity, ≥3 to <6 METs for moderate-intensity activity, and ≥ 6 METs for high-intensity activity. Moreover, we also determined the weekly number of steps and walking time.

### 2.4. Physical Activity Level

We calculated the physical activity level (PAL) as the TEE/basal metabolic rate (BMR). TEE was assessed with the triaxial accelerometer. BMR was calculated using the following equation for Japanese [14]:

(1) $$\begin{array}{c} \text{males: }\left\{\left(\text{0}.1238+\left(\text{0}.0481\times W\right)+\left(\text{0}.0234\times H\right)-\left(\text{0}.0138\times A\right)-\text{0}.5473\times 1\right)\right\}\times \frac{1000}{4.186},\\ \text{females: }\left\{\left(\text{0}.1238+\left(\text{0}.0481\times W\right)+\left(\text{0}.0234\times H\right)-\left(\text{0}.0138\times A\right)-\text{0}.5473\times 2\right)\right\}\times \frac{1000}{4.186}, \end{array}$$

where W = body weight in kg, H = body height in cm, and A = age in years.

The World Health Organization proposes that the BMR calculated using the above formulas can be used to classify the lifestyle patterns of subjects as very inactive [PAL <1.4], sedentary or lightly active [PAL ≥1.4 to <1.7], active or moderately active [PAL ≥1.7 to <2.0], or vigorous or vigorously active [PAL ≥2.00 to 2.40] [15].

### 2.5. Clinical Data Collection

Clinical data were collected from the hospital medical records. The nutritional condition was assessed using body mass index (BMI) and the controlling nutritional status (CONUT) score, which was determined using serum albumin, total cholesterol, and total lymphocyte count [16]. Patients with CONUT scores of 0-1 were considered to have a normal nutritional status; those with a score of 2–4 are at mild risk; a score of 5–8 indicates moderate risk; and a score of 9–12 reflected severe risk of malnutrition.

### 2.6. Statistical Analysis

All statistical analysis was conducted using the SPSS 20.0 for Windows (SPSS Inc., Chicago, IL). A *P* value <0.05 denoted the presence of a statistically significant difference. Descriptive statistics were presented as mean ±standard deviation. Normal distribution of data was assessed by the Shapiro–Wilk test (*P* > 0.05). Mann–Whitney's U test was used to identify significant differences between two groups. The Pearson correlation test was used to assess associations between PAL and age, BMI, ICD, MWD, ABI, and CONUT score in subjects with PAD and the Spearman correlation test was also used for the association between PAL and gender.

## 3. Results

The physical characteristics of the study participants are shown in Table 1. PAD subjects had a higher BMI, compared with age- and sex-matched control subjects. Eight of the 10 subjects with PAD (80%) reported IC symptoms during the test, and six (70%) could not complete the walking test due to severe pain. One subject had undergone peripheral vascular surgery and did not report symptoms of IC. Table 1 also shows the values of ABI, ICD, MWD, and CONUT score for the PAD group.

**Figure 1:**
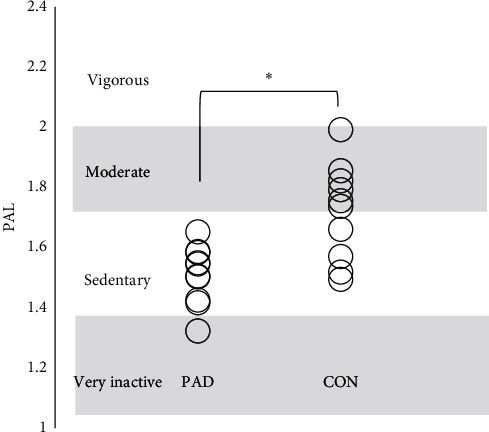
Classification and comparison of PAL between patients with PAD and control subjects (CON). ∗*P* < 0.001 between PAD and CON. PAL, physical activity level; PAD, patients with peripheral artery disease; CON, healthy control subjects.

The values of PAL, step counts, and the walking time were lower in PAD subjects than the control (Table 2, Figure 1). Assessment of physical activity of patients with PAL showed a mean PAL of 1.51 ± 0.97 and all subjects were classified as physically very inactive and sedentary. For the control subjects, the mean PAL was 1.75 ± 0.14, 27.3% of subjects were classified as sedentary, and 72.7% were classified as active. In both group, none was classified with vigorous activity (Figure 1).

**Figure 2:**
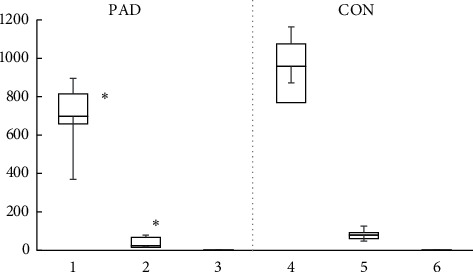
Box and whisker plots (median, quartile, and range) of time spent at activities of light-, moderate-, and high-intensity in PAD patients (left) and healthy control subjects (right). ^*∗*^*P* < 0.001 between PAD and CON at light- and moderate-intensity activities. PAD, peripheral artery disease patients; CON, healthy control subjects.

All subjects spent more time at low-intensity activities than moderate- or high-intensity activities.  Figure 2 shows the time spent on activities with light-, moderate-, and high-intensity for PAD patients and healthy controls. PAD subjects spent significantly less time in light- and moderate-intensity activity than the control subjects. However, there was no difference in the high-intensity activity (Figure 2). Furthermore, the total amount of time spent on all intensity physical activity in subjects with PAD was 71.3% of that in healthy control. Especially, the time spent at moderate-intensity activity was about 50% of that of healthy control. Subjects with PAD spent 40 fewer minutes walking each day (Table 2), with a daily step count that was also approximately 50% less compared to the control group.

In PAD subjects, there was no significant association between PAL and gender, ICD, MWD, ABI, and CONUT score, although a moderate correlation was identified between PAL and age (Table 3).

## 4. Discussion

This study investigated PAL of patients with PAD using triaxial accelerometer. The main findings were as follows: (i) patients with PAD had lower PAL than the control; (ii) there was no significant difference in the high-intensity activity between the two groups; however, the light- and moderate-intensity activities were significantly less in the PAD group than the control, especially the time spent at moderate-intensity activity was approximately 50% less (40 minutes fewer); and (iii) among patients with PAD, low PAL did not directly correlate with IC.

To investigate the cause of low physical activity in PAD patients, we selected PAL, which provides comprehensive evaluation of physical activities across all domains of daily life, as an exposure indicator. In this study, all participants with PAD were classified as very inactive or sedentary. As we expected, the mean PAL of subjects with PAD in the present study was significantly lower than that of the control (1.51 ± 0.97 vs 1.75 ± 0.14). A previous study of 99 healthy older men and women reported a mean PAL of 1.68 ± 0.28 [17]. Compared with the healthy subjects of the above study, a low mean PAL was also observed in our PAD patients. Thus, the present study demonstrated reduced amount of physical activity in patients with PAD.

Subjects with PAD spent 40 fewer minutes walking each day (Table 2). The lower PAL in the participants with PAD was primarily due to these shorter walking times at light- and moderate-intensity activities. Our results showed that the time spent at light- and moderate-intensity activities in patients with PAD was approximately 30% and 50% less than that of the control, respectively. The daily step count was also approximately 50% less in the PAD group than the control. Gardner et al. [18] compared patterns of physical activity between individuals with and without IC. They found that individuals with IC spent less time walking and took fewer steps, particularly at medium to high cadence levels. A high cadence level of walking is equivalent to moderate-intensity activity [19], and a moderate cadence level is considered to be equivalent to low-intensity activity. Thus, it is considered that their study supported our result. The greater differences in our present study are that our subjects are more than 10 years older and that we showed physical activity intensity. Walking in daily life is considered to require 2–4 METs and to be a moderate-intensity activity [20, 21]. Therefore, the lower mean PAL in the PAD group could be explained by spending less time in light- and moderate-intensity activities in daily life. In particular, the reduced amount of time walking was the main contributor to the lower mean PAL. It has also been shown previously that the time spent at low- and moderate-intensity activities determines the PAL and high-intensity activity does not have a significant impact on PAL [22–25]. Also, in view of the present result of the lack of difference in high-intensity activity, obtaining a higher PAL in PAD patients does not necessarily require performance of high-intensity activities such as sports.

Differences exist among previous studies with regard to the relationship between IC and physical activity [26]. This could be due to differences in leg symptoms and/or walking test. Although IC has long been considered the hallmark symptom of PAD, several studies reported that more patients with PAD are asymptomatic or have atypical leg symptoms than those with classic intermittent claudication [27–29]. We also expected that diminished physical activity in daily life was a consequence of IC, however it did not. For our patients with PAD, the ICD as the walking parameters were not associated with PAL (Table 3). This finding suggests inconsistent association between onset walking distance and PAL in PAD. It was reported that the limitation in exercise performance during standardized treadmill testing in PAD patients with atypical leg pain is vascular-mediated, similar to patients with claudication [28]. In addition, the limited walking and exercise ability in PAD seems to depend on complex interaction of various pathological factors, including arterial obstruction, endothelial dysfunction, and altered skeletal muscle phenotypes such as mitochondrial dysfunction and inflammation [29–31]. Therefore, ICD, as the walking distance, might not be reflected in PAL (Table 3). One important finding of this study is the fact that PAD limits the amount of physical activity regardless of leg symptoms. Some studies describe the relationship between physical activity and the distance walked during the 6-minute walk test in IC patients [32, 33]. This test is a submaximal exercise test used to assess aerobic capacity and endurance. Therefore, we would expect that individuals with higher levels of physical activity would be to cover longer distances in this time interval. However, since we examined a route of 20 meters with turns for only 12 laps, MWB at the present study did not reflect their aerobic capacity. For this reason, the present study did not show the relationship between physical activity and MWD.

In patients with PAD, low daily PAL is a strong predictor of mortality [34]. In this regard, a physically active lifestyle has the potential to modify peripheral artery risk factors and promote general health [5, 35, 36]. Considering the serious health risks associated with low mean PAL, one way to improve PAD patient's health is by increasing the amount of low- and moderate-intensity physical activity throughout the day. Moreover, it is important to recommend increasing the number of steps or walking time per day. Those variables are modifiable through rehabilitation and educational intervention. For patients with PAD, it is important to gain a better understanding of the characteristics of physical activities that contribute to their health. The present results showed that high-intensity activity does not have a significant impact on PAL in PAD patients and suggested the need for educational intervention on physical activity for such patients. It is well known that supervised exercise programs improve walking ability [37]. However, not only exercise training but also increasing amount of walk in daily life are important for PAD patients.

## 5. Limitation

Several limitations of this study should be acknowledged. First, the sample size is relatively small. Second, BMI was significantly higher in PAD subjects than controls. However, recent research reported that obesity is causally associated with PAD [38, 39]. Therefore, a higher BMI in participants with PAD in the present study may be a consequence of the following: since obesity increases the risk of PAD, that alone may have been its cause rather than the consequence of a lack of physical activity due to IC. Future studies may benefit from a larger sample size as well as a more homogenous study cohort.

## 6. Conclusions

Our findings could help improve PAL in patients with PAD. We demonstrated that PAD patients have limited amount of physical activity, especially in light and moderate intensity regardless of leg symptoms. Our results highlight the importance of walking in the daily life of PAD patients.

## Figures and Tables

**Table 1:** Subjects' characteristics.

	PAD	Control	*P* value
*n* (women/men)	10 (2/8)	10 (6/4)	0.23
Age	76.7 ± 7.5	74.3 ± 5.8	0.42
Weight (kg)	64.4 ± 7.8	55.2 ± 8.8	0.02
Height (cm)	162.4 ± 7.1	161.9 ± 7.4	0.88
BMI (kg/m^2^)	24.4 ± 2.7	20.9 ± 1.8	0.03
Presence of IC (%)	80	—	
Resting ABI	0.68 ± 0.12	—	
ICD (m)	227.0 ± 157.8	—	
MWD (m)	296.0 ± 156.3	—	
CONUT score	0.9 ± 0.74	—	

BMI, body mass index; ABI, ankle brachial index; ICD, intermittent claudication onset distance; MWD, maximum walking distance. Data are mean ± SD.

**Table 2:** Average durations of different intensity levels and walking parameters.

	PAD	Control	*P* value
Light activity (min/day)	704.8 ± 158.4	976.9 ± 175.2	0.000
Moderate activity (min/day)	38.5 ± 28.1	79.1 ± 25.5	0.003
High activity (min/day)	0.4 ± 0.6	0.8 ± 0.6	0.089
Total amount of activity (min/day)	743.7 ± 166.1	1004.2 ± 178.9	0.000
Step counts (steps/day)	3250 ± 1989	6055 ± 2085	0.006

Data are mean ± SD.

**Table 3:** Correlation matrix of the variables in PAD patients.

	PAL		Age	Gender		BMI		ABI	ICD		MWD
PAL											
Age	−0.689	^*∗*^									
Gender	0.087		0.306								
BMI	0.382		−0.109	0.174							
ABI	0.268		−0.118	0.696	^*∗*^	0.185					
ICD	0.104		−0.266	0.000		0.339		0.484			
MWD	0.21		−0.458	−0.443		0.195		0.116	0.836	∗∗	
CONUT score	0.539		−426	0.283		0.800	∗∗	0.573	0.503		0.285

^*∗*^ *P* < 0.05 (2 tailed). ^*∗∗*^*P* < 0.01 (2 tailed). PAL, physical activity level; BMI, body mass index; ABI, ankle brachial index; ICD, intermittent claudication onset distance; MWD, maximum walking distance; CONUT, controlling nutritional status score.

## Data Availability

The data used to support the findings of this study are restricted by the Ethics Committee in order to protect subjects' privacy. Data are available from Sumiko Shiba, for researchers who meet the criteria to access confidential data.
